# Treatment of myoclonic-atonic epilepsy caused by SLC2A1 de novo mutation with ketogenic diet: A case report

**DOI:** 10.1097/MD.0000000000015428

**Published:** 2019-05-03

**Authors:** Zihan Wei, Luojun Wang, Yanchun Deng

**Affiliations:** Department of Neurology, Xijing Hospital, Air Force Military Medical University (Fourth Military Medical University), Xi’an, People's Republic of China.

**Keywords:** case report, glucose transporter type 1 deficiency syndrome, ketogenic diet, myoclonic-atonic epilepsy, SLC2A1, whole exome sequencing

## Abstract

**Rationale:**

The SLC2A1 gene encodes glucose transporter 1 on blood–brain barrier, which plays an important role in the energy supply for neurons. Mutations in SLC2A1 gene can cause many clinical syndromes, including glucose transporter type 1 deficiency syndrome and many types of epilepsy syndromes such as childhood absence epilepsy and myoclonic-atonic epilepsy, etc. Ketogenic diet has been proved to be very effective on those cases. Clinically, SLC2A1 gene mutations are quite rare.

**Patient concerns:**

Repeated unconsciousness and bilateral limb weakness lasted for 3 years.

**Diagnoses:**

Myoclonic-atonic epilepsy.

**Lessons:**

After taking whole exome sequencing, we found out that there is a de novo insertion mutation in the patient's SLC2A1 gene, leading to frameshift. As a result, ketogenic diet (2:1, 4 times a day) was used as the treatment. As for the patient, total calories intake per day was controlled at 1190 kcal. The calories per kg per day were 66.11 kcal/kg. The amount of ketone bodies was controlled at 2 to 3 mmol/L and the concentration of plasma glucose was controlled at 4 to 5 mmol/L.

**Outcomes:**

After the launch of ketogenic diet, the patient has been seizure free for nearly a year and stopped all his antiepileptic drugs.

**Conclusion:**

Our case suggests that gene examination is very important part of the diagnosis of epilepsy etiology and epilepsy syndromes. Ketogenic diet should be considered as the first line therapy with SLC2A1 gene mutations.

## 1 Introduction

Normally, neurons in central nervous system need to use glucose as energy supply. Glucose transporter 1 (Glut1), located at the blood–brain barrier, guarantees the facilitated transport of glucose into the brain.^[1]^ Glut1 is coded by SLC2A1 gene, which locates on chromosome 1p34.2 and contains 10 exons.^[2]^ The first disease concerned Glut1 was described by De Vivo et al^[3]^ as Glut1 deficiency syndrome (Glut1-DS). Until now, there are <300 cases being reported worldwide and most of the reported cases are sporadic. The spectrum of Glut1-DS ranges from classical infantile epileptic encephalopathy to atypical forms such as paroxysmal dystonia, alternating hemiplegia, and migraine. So, the possibility of acquiring Glut1-DS should be considered in patients with intractable epilepsy.^[4]^ Considering the fact that patients with Glut1-DS often represent multiple antiepileptic drugs resistant, ketogenic diet (KD) has been proved to be the best choice.^[5]^

Here, we report a case of myoclonic-atonic epilepsy (MAE) caused by a novel de novo mutation in SLC2A1 gene. After using KD, the patient has been seizure free for nearly a year and gradually stopped taking anti-epileptic drugs (AEDs).

## 2 Case study

### 2.1 Clinical evaluation

A 4-year-old boy accompanied by his parents visited the outpatients department of our hospital because of repeated unconsciousness and bilateral limb weakness.

In 2014, when the patient was 1-year-old, he experienced his seizure for the first time. The epileptic fit lasted for about 10 minutes, mainly presenting as myoclonus, bilateral limb weakness, and unconsciousness. Similar fits occurred for another 3 times before he was taken to local hospital and diagnosed as epilepsy. Levetiracetam (0.25 g in morning, 0.375 g at night) was prescribed for oral administration. He responded positively to the therapy. The longest seizure-free period lasted for 2 years. In this period, his parents found that he walked unstably and his mental development was slower than children of the same age. In 2016, however, his seizure frequency rose to 3 times per month. The symptoms presented as short-term unconsciousness and sudden bilateral limb weakness. Sodium valproate oral solution (6 mL, twice a day) was added to his therapy. However, the application of sodium valproate made no differences. Then clonazepam (0.5 mg, twice a day) was added to the therapy too. With all these AEDs, the frequency of his onsets didn’t change.

He was referred to our hospital because the frequency of his seizures had risen to once a day. The 24 hours video EEG (VEEG) monitoring was taken. The VEEGs during wakefulness showed unsynchronized spike slow waves appearing for many times (Fig. 1 A). Generalized sharp slow waves were also found during patient's non-rapid eye movement sleep (Fig. 1 B). He doesn’t have history of intoxication, brain trauma, or central nervous system infections. The pregnancy was normal. His delivery was normal. According to his clinical manifestations and VEEG results, he was diagnosed as MAE. In order to find out the etiology of his epilepsy syndrome, we decided to take whole exome sequencing (WES) for the patient and his biological parents. We reviewed the WES report that the parents had already done in a third-party laboratory.

**Figure 1:**
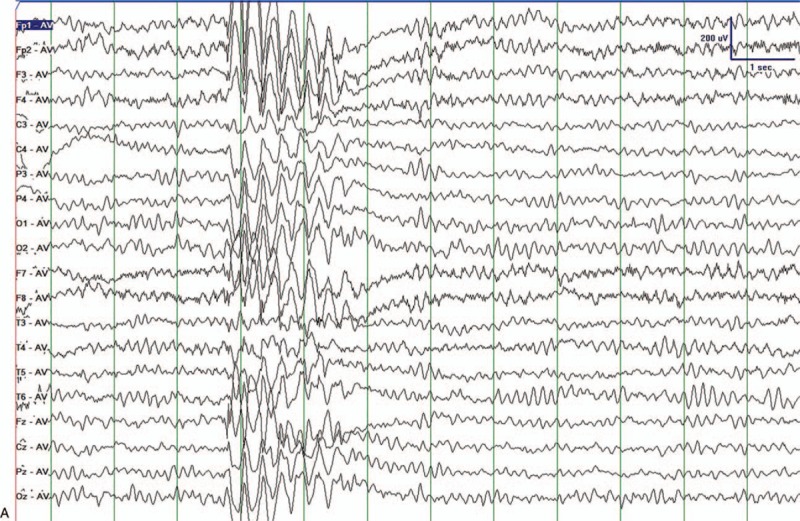
Clinical evaluation processes we took after receiving the patient. (A) VEEGs during wakefulness. Unsynchronized sharp and spikeslow waves appeared. (B) Sleeping VEEGs. Generalized spike and slow waves during NREM sleep appeared. VEGG = video electroencephalography.

**Figure 1 (Continued):**
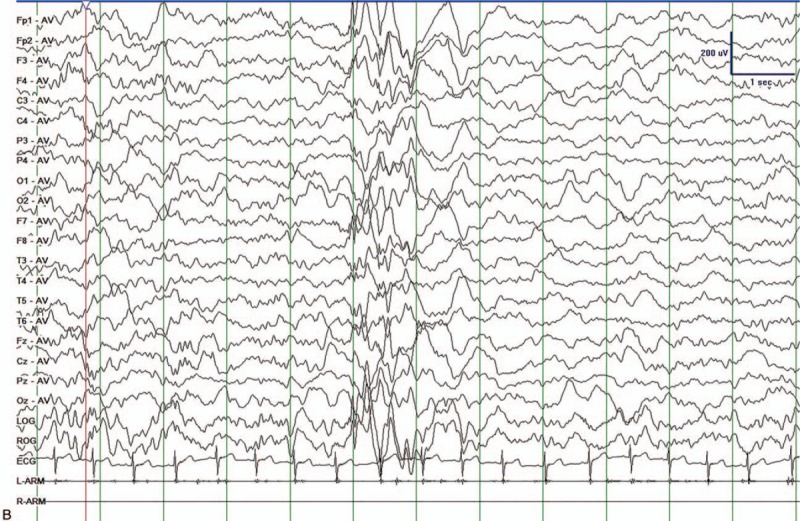
Clinical evaluation processes we took after receiving the patient. (A) VEEGs during wakefulness. Unsynchronized sharp and spikeslow waves appeared. (B) Sleeping VEEGs. Generalized spike and slow waves during NREM sleep appeared. VEGG = video electroencephalography.

### 2.2 The results of genetic analyses

**Figure 2:**
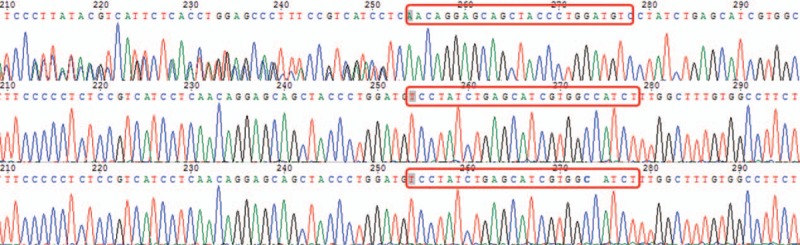
The first row belongs to the patient. Compared with standard sequence of SLC2A1 gene, there are a mutation in the patient's gene, a 25 bp insertion mutation between c1094 and c1095 in exom 9. There are no mutations in his parents’ exome. The red boxes indicate the insertion in the proband and normal sequences in his parents.

The WES revealed a de novo heterozygous insertion mutation in the patient's SLC2A1 gene. Sanger sequencing of the patient and his biological parents were performed. The insertion was in the exon 9 of solute carrier family, member1 gene, which is a 25 bp fragment (AACAGGAGCAGCTACCCTGGATGTC) between c1094 and c1095 (Fig. 2). Considering the fact that this mutation might cause serious misfunction of glucose transporter 1, the mutation was considered pathogenic according to the American College of Medical Genetics and Genomics guideline.^[6]^

### 2.3 Treatment and prognosis

**Figure 3:**
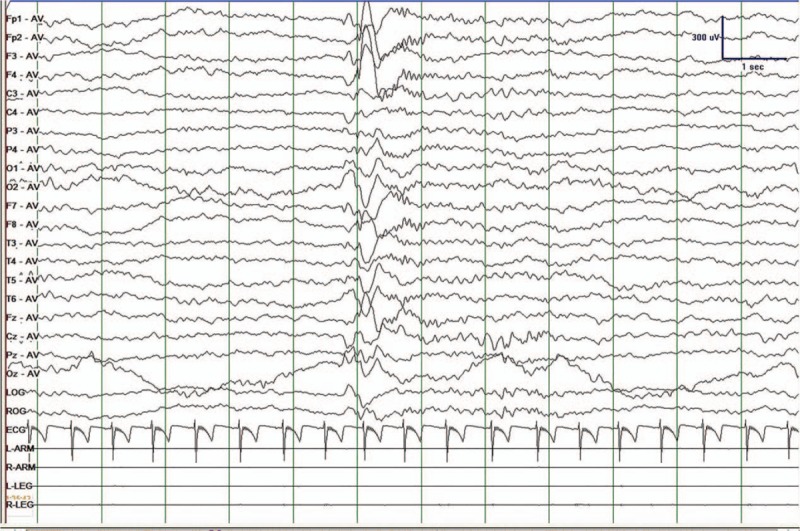
VEEGs results after ketogenic therapy for 6 months. Only few slow sharp waves appeared in his VEEGs. VEGG = video electroencephalography.

According to the result of WES and VEEGs of the patient, he was diagnosed as MAE caused by SLC2A1 gene mutation. Consequently, KD was used as the treatment. The first day after KD he had not experienced seizure. The patient was instructed to gradually decrease the amount of clonazepam and valporate. One month after KD, he stopped using clonazepam and valporate and started to decrease the amount of levetiracetam (0.25 g in morning, 0.375 g at night). By the end of this month, he stopped to take levetiracetam. He only uses KD (2:1, 4 times a day) as a treatment from 2017. As for the patient, total calories intake per day was controlled at 1190 kcal. The calories per kg per day were 66.11 kcal/kg. The amount of ketone bodies was controlled at 2 to 3 mmol/L and the concentration of plasma glucose was controlled at 4 to 5 mmol/L. The VEEGs results showed that slow sharp waves only appeared occasionally (Fig. 3). Until the last follow-up at June 25, 2018, his parents reported that he didn’t have seizures for nearly 1 year. His intelligence development was back to normal. The KD therapy had an excellent positive effect on both of his epilepsy and cognition.

## 3 Discussion

SLC2A1 locates in chromosome 1p34.2. The gene is 35 kb in length and contains 10 exomes. Hundreds of mutations have been found in Glut1-DS, including microdeletion, missense mutation, nonsense mutation, frameshift, and splice site mutation and most of these mutations mentioned above are de novo heterozygous mutations.^[7]^ These heterozygous mutations will result in haplo-insufficiency of Glut1. The transportation of glucose through blood–brain barrier will be affected by the haplo-insufficiency of Glut1 which will lead to low glucose level in patients’ cerebrospinal fluid. The low glucose level will lead to energy deficiency in their brain tissue, which may result in a series of neurological symptoms. In our case, the patient carries a rare frameshift mutation.

According to the clinical symptoms, Glut1-DS can be divided into classical type and nonclassical type.^[8]^ About 90% of the reported cases are related to the classical type of Glut1-DS. Its clinical symptoms are mainly developmental encephalopathy accompanied with infantile seizures. Classical type Glut1-DS patients usually suffer their first seizure before 2-year-old. The morbidity of Glut1-DS is at its highest point among age 1 to 6 months. The characteristics of the clinical seizures include generalized tonic-clonic seizures, myoclonus seizures, atypical absence seizures, atonic seizures, and focal seizures. The most common symptoms are myoclonus seizures and atypical absence seizures. The patients were born with a normal head circumference while the growth of his head circumference is slower than children of the same age. Patients with classical type also have cognitive impairment.^[3]^ They may also have symptoms suggesting impairment of pyramidal system, extrapyramidal system, and cerebellum.^[9]^ The patients suffered from nonclassical Glut1-DS usually presents paroxysmal ataxia, weakness, and dyskinesia with or without epilepsy.^[8]^ The discovery of SLC2A1 gene mutation is the most important diagnostic basis for Glut1-DS and provides specific therapy for this disease.

MAE, also known as Doose syndrome, is a rare kind of epilepsy syndrome. MAE accounts for 1% to 2% of epilepsy in children. 24% of patients had febrile convulsion before 1-year-old and most of patients are men. A recent French multicenter retrospective study^[10]^ indicated that the early use of KD in the course of MAE can have strong antiepileptic effect. The patients may have a better cognitive result by early using KD. However, gene examination was not included in this study.

The patient discussed in this case had a rare and long 25 bp insertion mutation in SLC2A1 gene. This mutation is a novel de novo pathogenic mutation for Doose syndrome. The discovery of the mutation indicates the importance of genetic diagnosis for epilepsy etiology, especially for Doose syndrome. With the results of gene diagnosis, we treated the patient with KD and gradually withdrew all the AEDs. He got seizure free for nearly 1 year. The patient's treatment with KD not only reduced the financial burden of patients’ family, but also avoided side effects of these drugs he had taken. KD therapy not only can alleviate epilepsy but also recovered the cognitive ability of the patient. This case may be considered as a typical example of precision medical treatment for epilepsy syndromes.

## Acknowledgments

The authors greatly appreciate the patient who volunteered to participate in the experiments described in this paper.

## Author contributions

Yanchun Deng collected and analyzed the data. Luojun Wang and Zihan Wei were joint first authors. All authors read and approved the final manuscript.

**Resources:** Yanchun Deng.

**Writing – original draft:** Zihan Wei, Luojun Wang.

**Writing – review & editing:** Yanchun Deng.

## References

[1] Thorens B Mueckler M Glucose transporters in the 21st Century. Am J Physiol Endocrinol Metab 2010;298:E141–5. 20009031 10.1152/ajpendo.00712.2009 PMC2822486

[2] Escary J Cecillon M Maciazek J Mutational analysis of GLUT1 (SLC2A1) in glut-1 deficiency syndrome; dong wang; pamela kranz-eble; darryl C. De vivo; (Article was originally published in human mutation 16:224-231, 2000). Hum Mutat 2000;16:527. 11102982 10.1002/1098-1004(200012)16:6<518::AID-HUMU9>3.0.CO;2-Q

[3] De Vivo DC Trifiletti RR Jacobson RI Defective glucose transport across the blood-brain barrier as a cause of persistent hypoglycorrhachia, seizures, and developmental delay. N Engl J Med 1991;325:703–9. 1714544 10.1056/NEJM199109053251006

[4] Klepper J GLUT1 deficiency syndrome in clinical practice. Epilepsy Res 2012;100:272–7. 21382692 10.1016/j.eplepsyres.2011.02.007

[5] Koch H Weber YG The glucose transporter type 1 (Glut1) syndromes. Epilepsy Behav 2019;91:90–3. 30076047 10.1016/j.yebeh.2018.06.010

[6] Richards S Aziz N Bale S Standards and guidelines for the interpretation of sequence variants: a joint consensus recommendation of the American College of Medical Genetics and Genomics and the Association for Molecular Pathology. Genet Med 2015;17:405–24. 25741868 10.1038/gim.2015.30 PMC4544753

[7] Liu Y Lee J Bellows S Evaluation of non-coding variation in GLUT1 deficiency. Dev Med Child Neurol 2016;58:1295–302. 27265003 10.1111/dmcn.13163

[8] Wang D Pascual JM De VD Adam MP Ardinger HH Pagon RA Glucose transporter Type 1 deficiency syndrome [updated 2018 Mar 1]. GeneReviews [Internet]. Seattle, WA: University of Washington, Seattle; 2002;1993–2019. 20301603

[9] Roser P Abbie C Michael R The spectrum of movement disorders in Glut-1 deficiency. Mov Disord 2010;25:275–81. 20063428 10.1002/mds.22808

[10] Stenger E Schaeffer M Cances C Efficacy of a ketogenic diet in resistant myoclono-astatic epilepsy: a French multicenter retrospective study. Epilepsy Res 2017;131:64–9. 28273610 10.1016/j.eplepsyres.2017.02.005

